# Activities and Interests of the Buffalo Academy of Medicine1Presidential address at the annual meeting held June 9, 1908.

**Published:** 1908-09

**Authors:** Allen A. Jones

**Affiliations:** Buffalo, N. Y.


### Activities and Interests of the Buffalo Academy of Medicine.[1]

By ALLEN A. JONES, M.D., Buffalo, N. Y.

IT becomes my pleasant office to address you in reference to the activities and interests of the Academy of Medicine at this its annual meeting. So efficient has been the work of the officers of this organisation in preceding years that but few avenues of advance and achievement seemed open when the year began. Fortunately, however, the president of the Academy found himself associated with so capable, zealous and industrious a body of co-workers in the council and general membership that the task of upholding the traditions of the Academy and of furthering its good work was made easier. The officers of the various sec-

1. Presidential address at the annual meeting held June 9, 1908.

tions prepared with commendable despatch excellent programs for the ordinary weekly meetings and also for the stated meetings. All sections have been well attended throughout the year and your president embraces this opportunity of expressing his highest sense of appreciation of the unflagging interest the section officers have shown during the past season, of the fine order of the work accomplished in their sections, and of the efforts they have made to increase the attendance at the meetings and to stimulate the coöperation of all the members of the Academy in the scientific, the ethical, the social work of our organisation. The spirit of good fellowship that has made itself manifest in our midst, quite precludes any but the kindliest feelings and good sense of justice when especial attention is called to the unprecedented attendance at some of the meetings of our surgical section. With unselfish sacrifice of time and other things involved by absence from home, eminent members of our profession from other cities have contributed to our pleasure and profit by notable scientific presentation at our meetings. At this the closing meeting of the year it seems fit to express anew the deep sense of appreciation of the Academy for the presence and contributions of our guests.

Pray let me here voice my gratitude to the chairmen and members of the special committees reporting tonight. I am not unmindful of the onerous duties imposed upon them nor of the generous manner in which they have discharged such duties. It falls within the province of physicians to interest themselves intimately with questions affecting the health and welfare of the community, and it was this thought that prompted me to appoint the several commissions that report this evening. To the members of the council individually and collectively allow me to express my thanks for their kindly and courteous and faithful cooperation and help. We have had ten council meetings that have served to demonstrate, among other things, that frank independence of opinion is not incompatible with effective collective work.

Also recognition of the hard work, well performed, of our able secretary is fitting here. The extraordinary labors that have been imposed upon him this past year furnish an evidence of the activity of our organisation and the Academy has been well served throughout. I wish to make public acknowledgment of the courteous assistance our secretary has at all times extended to me.

The committee on the Academy building has found it impossible to accomplish more than to engage the sympathetic interest of the Academy in the acquisition of its own home. Progress, however, has been made and in due time, I have no doubt, we will have a suitable building in a suitable situation. Our financial position grows stronger each year and eventually no burden whatever will be imposed upon us by an acquisition of a reasonable property. But even in the event of an Academy building becoming a slight obligation is that a sufficient reason for abandoning so laudable a project? It certainly is not in my view. On the contrary, it should serve as a stimulus for greater and better achievements. What are difficulties for in this world if not to increase our efficiency and raise us higher in our activities, our perseverance and our unselfish devotion to worthy ends. We are agreed as to the desirability of progress in the life and work of the Academy; we should allow no minor considerations to turn us away from a laudable project. Our interests will be notably advanced by surmounting some difficulties. We all face obstacles and perplexities daily but we do not turn away from them; let us be true to our higher aims. We ought to have our own building of simple but classic and dignified construction. We want that building to symbolise scientific progress and ambition. We want it to be the home and center of a larger, better spirit in the profession, free from petty jealousies and rivalries. We want it to foster comradeship and good-fellowship in our profession. We want it because we are co-workers, because our interests are one, because in unity there is happiness as well as strength. We want it to dispel discouragement and to exemplify that while many things crumble away institutions live. We want it because it will represent a fitting accomplishment, obstructions thrown aside and difficulties vanquished. We want it because it is eminently appropriate that the medical fraternity in this great city should have a temple of its own and because it will serve to illustrate our devotion to the art and science and literature of medicine. If the time is not yet here, if it must be postponed for awhile do not let the higher possibilities of the Academy grow distant and faint upon the horizons of your memories.

436 Franklin Street.

